# The D*Phase-study: study protocol for a pragmatic two-phased, randomised controlled (non-inferiority) trial that addresses treatment non-response and compares cognitive behavioural therapy and short-term psychodynamic supportive psychotherapy for major depression

**DOI:** 10.1186/s12888-021-03156-8

**Published:** 2021-05-04

**Authors:** M. F. Miggiels, P. M. ten Klooster, S. Bremer-Hoeve, J. J. M. Dekker, M. J. H. Huibers, E. Reefhuis, H. L. Van, M. K. van Dijk

**Affiliations:** 1 Dimence, Deventer, The Netherlands; 2 Department of Clinical Psychology, Vrije Universiteit Amsterdam, Amsterdam, The Netherlands; 3 Universiteit Twente, Enschede, The Netherlands; 4 ARKIN, Amsterdam, The Netherlands; 5 NPI Center for Personality Disorders, ARKIN, Amsterdam, the Netherlands

**Keywords:** Depression, Psychodynamic psychotherapy, Cognitive behavioural therapy, Prescriptive factors, Non-response, Working alliance, Allegiance, Treatment integrity

## Abstract

**Background:**

Several evidence-based psychotherapeutic treatment options are available for depression, but the treatment results could be improved. The D*Phase study directly compares short-term psychodynamic supportive psychotherapy (SPSP) and cognitive behavioural therapy (CBT) for Major Depressive Disorder (MDD). The objectives are 1. to investigate if, from a group level perspective, SPSP is not inferior to CBT in the treatment of major depressive disorder, 2. to build a model that may help predict the optimal type of treatment for a specific individual; and 3. to determine whether a change of therapist or a change of therapist and treatment method are effective strategies to deal with non-response. Furthermore (4.), the effect of the therapeutic alliance, treatment integrity and therapist allegiance on treatment outcome will be investigated.

**Method:**

In this pragmatic randomised controlled trial, 308 patients with a primary diagnosis of MDD are being recruited from a specialised mental health care institution in the Netherlands. In the first phase, patients are randomised 1:1 to either SPSP or CBT. In case of treatment non-response, a second phase follows in which non-responders from treatment phase one are randomised 1:1:1 to one of three groups: continuing the initial treatment with the same therapist, continuing the initial treatment with another therapist or continuing the other type of treatment with another therapist. In both treatment phases, patients are offered sixteen twice-weekly psychotherapy sessions. The primary outcome is an improvement in depressive symptoms. Process variables, working alliance and depressive symptoms, are frequently measured. Comprehensive assessments take place before the start of the first phase (at baseline), in week one, two and four during the treatment, and directly after the treatment (week eight).

**Discussion:**

While the naturalistic setting of the study involves several challenges, we expect, by focusing on a large and diverse number of research variables, to generate important knowledge that may help enhance the effect of psychotherapeutic treatment for MDD.

**Trial registration:**

The study was registered on 26 August 2016 with the Netherlands Trial Register, part of the Dutch Cochrane Centre (NL5753), https://www.trialregister.nl/trial/5753

## Background

According to estimates of the World Health Organization, 4.4% of the global population suffered from depression in 2015 [1]. Given the high prevalence of major depressive disorder and its severe impact on the functioning of patients who suffer from it, it is not surprising that depression is one of the three leading causes of the global disease burden [2]. Fortunately, several evidence-based pharmaco- and psychotherapeutic treatment options are available. Research suggests that most patients suffering from a psychiatric disorder prefer psychotherapy over medication [3]. Numerous well-conducted studies have demonstrated the effectiveness of several kinds of psychotherapy for MDD [4]. Although cognitive behavioural therapy (CBT) has been studied most [5, 6], there is no robust evidence indicating that efficacy varies between CBT and other evidence-based types of psychotherapeutic treatment for MDD such as interpersonal psychotherapy, behavioural activation, problem-solving therapy and psychodynamic psychotherapy [7–9]. The fact that the outcome estimates of different kinds of psychotherapy for MDD seem to be quite similar has led some people to argue that the effects of the different available psychotherapeutic treatment methods can mainly be attributed to common factors [10–12]. Common or non-specific factors are thought to be universal in all psychotherapy rather than specific to the particular method used. On the other hand, there are strong arguments against this common-factor hypothesis. See for a comprehensive overview Cuijpers, Reijnders and Huibers [13]. The presumed equivalence of different forms of psychotherapy therefore gives rise to an important debate about the possible working mechanisms underlying psychotherapy for depression.

The availability of so many different evidence-based psychotherapeutic treatments for depression that at least appear to yield comparable treatment results can nevertheless be viewed as a good thing. It means that there are several viable options available to which the therapist can resort if response is insufficient, particularly if one considers that both patients and therapists may have different preferences for particular types of treatment.

Worldwide, a substantial number of psychotherapists practise psychotherapy from a psychoanalytic perspective [14, 15]. The first major objective of the present study is to add to the evidence base for Short-term Psychodynamic Supportive Psychotherapy (SPSP) [16, 17]. SPSP is a specific form of psychodynamic psychotherapy that has been tested in several RCTs in the Netherlands [18] and was found to be non-inferior to Cognitive Behavioural Therapy (CBT) for patients with major depressive disorder [19]. The first part of the study consists of an RCT in which patients are randomly assigned to Short-term Psychodynamic Supportive Psychotherapy (SPSP) or Cognitive Behavioural Therapy (CBT).

The empirically established overall effectiveness of psychotherapy does not imply that all patients will benefit from treatment. A meta-analysis of the treatment outcomes of evidence-based forms of psychotherapy estimates that the number of patients that does not respond (as defined by an improvement of 50% or more on any given self-report depression-severity scale) is substantial (52%), while 57 to 59% do not achieve full remission after one treatment option [4] (as defined by a cut-off score on a depression-severity scale [20]). This implies an improvement in the treatment results in psychotherapy for depression is urgently needed. But where to begin? The development of new forms of psychotherapy over the years does not seem to have made treatment more effective [21]. One cannot therefore expect this to be automatically changed by the introduction of SPSP as a new form of evidence-based treatment for depression. The proponents of the ‘common factor’ hypothesis would also recommend focusing more on researching and working on the optimisation of non-specific treatment factors.

Another important approach in the quest to improve the results of psychotherapy would be to focus on the possibility that patients with specific characteristics respond differently to specific kinds of treatment; in other words, to learn more about which type of works best for which patients [22, 23]. If a specific method turns out to work better for specific patients, this would indicate that the use of a particular method probably *does* matter.

### Personalised therapy using a personalised advantage index

Studies of the use of a PAI (Personalised Advantage Index) found that, if patients receive indicated treatment in accordance with the PAI, there is a relevant and significant difference in treatment effect [24–26]. In this approach, the effect of one kind of psychotherapy for a specific individual by comparison with another kind of psychotherapy is predicted on the basis of an algorithm that is developed using advanced statistical methods [25, 27]. However, the utility of algorithms in clinical practice has not been established because the models that predict the best possible treatment method for individuals in a specific sample have not yet been validated. A PAI can be built only by using data from an RCT comparing two active treatments [28, 29]. CBT and SPSP are good candidates for a personalised predictive approach of this kind because they differ in several ways. They are located at either end of a spectrum with respect to the focus of therapy (symptom-centred to more person-centred). The stated mechanisms differ (for example, CBT is thought to change maladaptive cognitions and behaviours, whereas SPSP is thought to be more person-centred), as does the level of structure that is applied during the sessions (CBT is structured and SPSP is not). Lastly, only CBT uses homework assignments.

The second major objective of the study presented here is therefore to build a model that may help predict the optimal type of treatment for a specific individual.

### Non-response and therapy switch

At present, despite the prospect of improving treatment outcome if valid prediction models for personalised treatment selection become available, clinical practitioners are still faced with the difficult problem of patients not responding to treatment. Nevertheless, mainly on the basis of expert opinion rather than evidence, several guidelines for the treatment of depression provide recommendations for how to proceed in cases of non-response. For instance, in the United States, the advice of the American Psychiatric Association in its practice guideline for the treatment of patients with major depressive Disorder [30] is to intensify psychotherapy; to consider a switch to another psychotherapy; or switching to or adding antidepressant medication. The recommendations in the Dutch [31] guidelines are confined to switching to another kind of psychotherapy or antidepressant medication. The United Kingdom guidelines of the National Institute for Health Care and Excellence advise combining treatments (antidepressant medication and CBT) after initial non-response [32]. One recent study does indeed conclude that adding antidepressant medication or CBT to initial monotherapy (CBT or medication) leads to an increased response rate [33]. However, there is no research that supports the advice to switch to another type of psychotherapy when the patient prefers continuing psychotherapy [34]. A switch of therapy may require a switch of therapist because the initial therapist does not have the necessary expertise, but possibly also because a change in clinical stance is necessary, which could be less credible if delivered by the same person [35]. In line with the claims made by the advocates of the common-factor theory, the switch to another psychotherapist could in itself make a positive treatment response more likely. The patient could, for example, benefit from a change and establish a better working alliance with a new psychotherapist.

To our knowledge, the study presented here is the first to include a second treatment phase for non-responders to psychotherapy for MDD that addresses the possible effect of a switch of type of psychotherapy *and* therapist. Non-responders to therapy from the first treatment phase are randomised to three different groups (continuing with the same therapist and the same therapy, switching to another therapist for the same therapy, and switching to another psychotherapist and the other therapy). The third and last major objective of this study is to determine whether a change of therapist or a change of therapist and treatment method are effective strategies to deal with non-response. If the second of these two different strategies proves to be most effective, this could also serve as additional evidence for the relevance of the specific method used. At the least, knowledge about the effectiveness of different psychotherapeutic strategies to deal with initial treatment non-response can be expected to help improve treatment outcome. Even if the strategies studied fail to show any effect, then it will at least be clear that it might be best to switch to a different treatment modality altogether.

### The role of the working alliance

A minor objective of the D*Phase study will be to focus on the relevance of the therapeutic alliance as a predictor for treatment outcome. The alliance is commonly defined as the emotional bond established in the therapeutic dyad and the agreement between the therapist and patient about the goals of therapy and the steps necessary to achieve them [36]. The alliance has been repeatedly shown to be both positively and significantly correlated with treatment effect in different treatment methods [37]. This has often been interpreted as evidence that the therapeutic alliance is an important “common factor”. However, good alliances may just as well be the *result* of changes in symptoms (particularly early changes), rather than a cause [38]. Causal inferences can only be made if the mediator (in this case, the therapeutic alliance) *precedes* the treatment effect. There is a scarcity of methodically robust studies that meet this temporality criterion [39].

This study will therefore focus on a number of questions relating to the effect of the working alliance on treatment effect. To start with, how do symptom changes affect the working alliance? Does early symptom change predict the quality of the working alliance as perceived by the patient? And does the alliance also have an effect on symptom change in itself, as the common factor theory predicts?

In addition, there are other questions concerning the possible influence of the alliance. For example, does the alliance depend on the characteristics of the therapist, the patient or the match between them [38, 40–42]? The present study also represents a unique opportunity to examine whether, in the case of treatment non-response after treatment phase one, the effect of a switch of therapist or the switch of therapist *and* treatment method is mediated by a change in the therapeutic alliance. Should this be the case, that finding can serve as evidence that the quality of the therapeutic alliance is a causal factor in achieving good treatment outcomes.

### Treatment integrity and allegiance

Finally, in the quest to identify the factors that are the most relevant targets for the purposes of improving the outcomes of psychotherapy, this study will also focus on the concepts of treatment integrity and allegiance since therapist-related factors may also play a role in the treatment effect.

*Treatment integrity* consists of two factors: the therapist’s ability to apply therapy-specific techniques as intended (quality or competence) and the extent to which these techniques and methods are applied (quantity or adherence) [43–45]. A recent meta-analysis [46] of the influence of adherence and competence on treatment outcome across a wide range of mental disorders shows that adherence and competence generally have no significant effect. However, studies that specifically focus on the treatment of depression have found that the competence of the practitioner had a modest effect and reported a trend towards a positive effect of a larger degree of adherence.

The concept of *allegiance* is usually used in a scientific context, where it relates to the loyalty of a principal investigator to a specific treatment method [47]. In the current study, we will focus on the concept of allegiance as it relates to the *therapist* and its possible effect on the outcome of psychotherapy*.* There is only limited amount of empirical research into the effects of researcher allegiance on the outcome of RCTs, but meta-analyses indicate that allegiance has a clear effect on study outcomes [48]. It is plausible that, if allegiance affects the outcome of an investigation, similar effects will also be found in the treatment room [49]. The sparse experimental research that has been done may indicate that a therapist delivers treatment better when he or she has confidence in the particular type of therapy and when he or she is in favour of the underlying principles [50]. If this hypothesis is correct, it would certainly be worthwhile to investigate whether optimal matching between the indicated therapy and the practitioner who implements it could improve treatment results [51].

## Methods

### Design

**Fig. 1:**
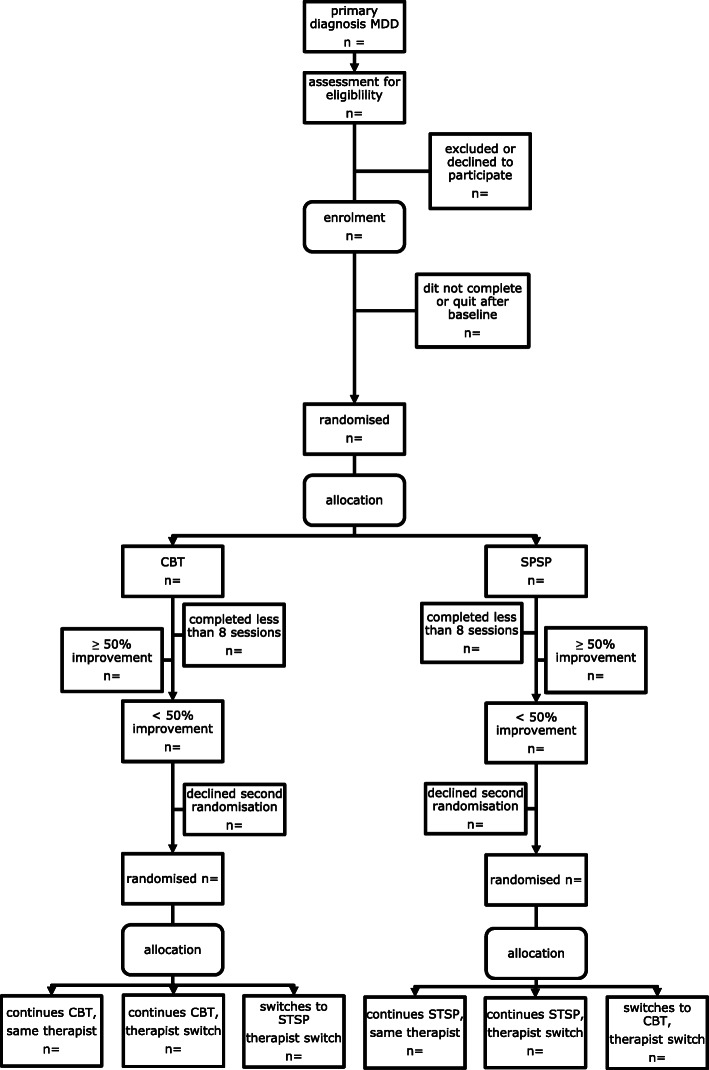
Participant flowchart

Patient inclusion for this study began in September 2016. The study is a large RCT involving the direct comparison of two treatments (Short-term Psychodynamic Structured Psychotherapy; SPSP; and Cognitive Behavioural Therapy; CBT). Before the eight-week period of the first phase of treatment, subjects are randomly assigned 1:1 to two groups and are offered 16 twice-weekly sessions of SPSP or CBT. In the second part of the study, patients who respond inadequately to treatment (defined by < 50% improvement on a depression severity scale) are randomly assigned 1:1:1 to one of three conditions: 1. continuing the initial treatment with the same therapist (control group), 2. continuing the initial treatment with another therapist, and 3. a switch of method *and* therapist; for a maximum of another 16 twice-weekly sessions. A participant flowchart is presented in Fig. 1. At baseline, patients complete several questionnaires that assess potential predictive factors. During the treatment, patients are asked to complete questionnaires about depressive symptoms, the working alliance and perceived therapist characteristics at several time points. In case of response, patients are asked to fill in follow-up questionnaires in week four and eight after completing treatment phase one. An overview of measures at different time points can be found in Table 1.

**Table 1:** Overview of variables, measures and time points

		Time points (week)											
		Intake	Baseline	Treatment (phase 1) week				Treatment (phase 2) week				Follow-up (phase 1 responders) week	
**Variable**	**Instrument**			**1**	**2**	**4**	**8**	**1**	**2**	**4**	**8**	**4**	**8**
Psychiatric diagnosis	MINI	•											
**Outcome variables**													
Depressive symptoms (primary)	IDS-SR		•	•	•	•	•	•	•	•	•	•	•
Functional impairment	SDS		•				•				•		•
Well-being	MHC-SF		•				•				•		•
**Predictors**													
Demographic variables			•										
Traumatic life events (recent)	LTE		•										
Traumatic life events (lifetime)	LEC-5		•										
Psychological symptoms	BSI	•											
Agoraphobia	AGO		•										
Self-esteem	RSES		•										
Rumination	RRS		•										
Defence style	DSQ 40		•										
Personality organisation	IPO		•										
**Therapist & alliance**													
Therapist characteristics	CRF-S			•				•					
Working alliance	WAI			•	•	•	•	•	•	•	•		

### Power and sample size calculation

To calculate the required sample size for the first phase of the study we used the original dataset provided by the authors of a similar study which was conducted in a similar setting and population [19]. We expected this to be the best data available to make an accurate estimate of the standard deviation of the primary outcome needed to calculate the sample size.

The primary outcome measure of the current study is the severity of depressive symptoms as measured by the IDS-SR [52]. To test whether SPSP is in fact (as hypothesised) not inferior to CBT, a treatment that has already proven to be effective for treating MDD, we used the same non-inferiority margin (the maximum allowed difference in the mean outcome scores between the treatment groups to conclude that SPSP is indeed non-inferior to CBT) for continuous measures as Driessen et al. [19, 53] Accordingly, we adopted a non-inferiority margin of five points on the mean score on the IDS-SR immediately after treatment. This margin of a five-point difference between SPSP and CBT corresponds to a Cohen’s d of .30, which we assume to be a clinically acceptable difference. Power analysis for continuous outcomes [54] indicated that, to prove that SPSP is not inferior to CBT, 268 patients in total are needed for the first part of the study to provide 80% power in order to demonstrate that the lower limit of the one-sided 95% confidence interval (CI) will be above the non-inferiority limit of − 5 for the IDS-SR (1-β = 80%, SD = 16.42). Given the observed dropout rate of around 15% for the first 100 included patients, the aim is to include 308 patients for the performance of the per-protocol analysis.

Assuming that approximately 52% of the patients will be classified as non-responder after the first phase of study [4], we estimate that around 139 patients can be randomized across the three groups (1. same therapist, same treatment, 2. a switch of therapist only, 3. a switch of therapist and treatment) in the second phase of the study. According to a sensitivity analysis, by means of an analysis of covariance (ANCOVA), this suggests that we can significantly (α = .05) demonstrate a medium to large effect size (f = 0.35) with 80% power between the three groups in the second phase of the study.

### Participants

All participants are referred outpatients at one of four locations of Dimence, a specialised mental health care institution in the northeast of the Netherlands. Most patients are referred by their GP and have, after a short screening, been assigned to one of the departments that specialises in treating adults (between 18 and 65 years old) suffering from anxiety and mood disorders. The participants are recruited from the group that meets the criteria for single or recurrent, moderate or severe episodes of unipolar major depression (Diagnostic and Statistical Manual of Mental Disorders, fourth edition, (DSM-IV [55], 296.22, 296.23, 296.32, 296.33) as classified as the primary diagnosis by the MINI international neuropsychiatric interview [56, 57]. During the study, DSM-5 [55] has been introduced in the Netherlands. Patients diagnosed with the condition that was previously classified as dysthymia and patients with depressive symptoms that persist for less than 2 months after the death of a loved one are therefore not included in the study, despite the fact that, according to DSM-5, they would meet the criteria for a depressive episode.

Patients can be included only if no psychotherapeutic treatment in specialised mental health care has been provided in the previous year. Exclusion criteria are: insufficient mastery of the Dutch language, in so far that this means the patient is unable to complete the necessary research questionnaires; psychotic symptoms; substance dependency (with the exception of nicotine dependency); not being able to commit to treatment requirements (such as following treatment sessions) and severe risk of suicide requiring immediate intervention. Patients taking antidepressants are included only after taking the same dose of medication for at least 6 weeks. If these patients that are using medication are willing to participate, they are told that the dosage and the medication should not be changed during the course of the study. Changes in medication are allowed only when strictly necessary, for example because of hazardous side-effects. The use of medication is monitored during the course of the study.

### Procedures

Psychologists, psychiatrists and nurses taking part in the intake assessment are asked to inform patients who meet the inclusion criteria about the nature of the study. Patient leaflets about the study are provided and, if necessary, a member of the research team is available to answer any questions. If the patient wishes to participate and does not meet the exclusion criteria, he or she is asked to sign an informed consent form in the days or weeks after the intake procedure. For the purposes of stratified randomisation, the person performing the initial intake assessment provides a member of the research team with information about the duration of the depressive episode (longer or shorter than 2 years). After the completion of the baseline measurement, patients are randomly assigned to one of two conditions (SPSP or CBT). For an overview of the questionnaires included during baseline and the treatment phases, see Table 1. All questionnaires are completed on an online platform. Therapists and researchers are blind to all outcomes except non-response (yes or no) after phase 1 of treatment. After phase 1 of treatment and approximately 16 sessions planned over a period of 8 weeks of treatment, the researcher is informed whether the patient has responded to treatment (yes or no). Response is defined as an improvement of at least 50% of the post-treatment IDS-SR score by comparison with the baseline assessment. Patients who do not respond to treatment are randomly assigned to one of three different treatment conditions for the second phase, as described above.

### Randomisation

We use two potential moderators of outcome: severity (IDS-SR score < 39 or ≥ 39) and duration of depression (longer or shorter than 2 years) as stratification variables. Randomisation takes place using a system with closed envelopes in which the researcher is kept blinded to treatment allocation until the completion of the study phase and the patient is blind to the research condition until the start of the therapy. The therapist is given the information about the type of treatment to be administered shortly before the start of therapy.

Randomisation lists with an 1:1 allocation ratio were drawn up prior to the start of the inclusion of patients using an online tool [58]. Random block sizes are used. Block randomisation for phase two follows the same procedure as phase one, with an allocation ratio of 1:1:1. Patients receive a unique randomisation code that is linked to a unique patient number in the data file. The coding key is known to only one independent person. That person manages the data file that will be used for statistical analysis.

### Interventions

#### Short-term psychoanalytic supportive psychotherapy

Short-term Psychoanalytic Supportive Psychotherapy (SPSP) was developed by de Jonghe [16, 17]. SPSP is characterised by an open dialogue that allows the therapist to be as responsive as possible to relevant issues brought up by the patient. The therapy is based on a relational perspective relating to six psychoanalytic sub-theories: drift theory; egopsychology; object relationship theory; self-psychology; attachment theory and primary-love theory. The focus of the treatment is primarily on the affective, and if necessary behavioural, aspects of the client’s functioning in current relationships. It is assumed that past relational experiences continue to affect adult relationships and are related to the onset of depressive reactions. SPSP is a more supportive variant of short-term psychoanalytic psychotherapy, which means a secure helping alliance is considered to be a prerequisite. Expressive interventions to enhance insight into actual relational patterns or earlier experiences are always embedded in a supportive context. The most important therapeutic technique is adequate psychoanalytic support [17, 59]. This is present when developmental needs that are not adequately met during life are (in part) experienced within the therapeutic relationship. Adequate psychoanalytic support is present when there is a supportive attitude that is not only intended by the therapist but also experienced as such by the patient and promotes personal growth.

In order to enhance progress and help to find a focus, the SPSP sessions can be structured on the lines of eight different levels of discourse [17]. Levels one, two and three focus in succession on the patient’s physical and psychological symptoms and complaints, the influence of life circumstances on the depressive symptoms, and the influence of external interpersonal relationships on the depressive symptoms. At the fourth and fifth level the focus shifts to one or more relational patterns in the patient’s life and the patient’s attitude in life, respectively. The sixth level works on how past relationships persist in the patient’s current life, and the seventh level addresses the intrapersonal relationship the patient maintains with himself or herself as a consequence of identification with these past relationships. At the eighth level the focus shifts to how the problems discussed at levels four to seven manifest themselves in the relationship with the therapist. The levels of discourse can vary considerably during the course of treatment. Short Psychodynamic Supportive Psychotherapy has been described by de Jonghe in a treatment protocol [60] which is used in the current study.

#### Cognitive behavioural therapy

Cognitive Behavioural Therapy (CBT) was developed by Beck [61] and it is based on the cognitive theory. The cognitive component of CBT aims to locate and correct negative automatic thoughts and logical errors, and to change ‘schemata’ and therefore alleviate the depressive symptoms. In addition to this cognitive element, CBT has a behavioural component that is based on the notion that depression is partly caused or maintained by a lack of pleasant or satisfactory activities in line with the theory of Lewinsohn [62]. In CBT, patients are therefore encouraged to identify activities that affect their mood positively and engage in them more often. CBT in general is further characterised by a limited time span and a structured approach. The focus of therapy is mostly on the present. Homework assignments are an important part of the treatment and patients use a workbook to register assignments. CBT is delivered in accordance with an existing protocol [63] and it is based on the principles for treating depression described by Beck [61] and the behavioural model described by Lewinsohn [64].

### Assessments

#### Primary outcome measure

##### Depression: inventory of depressive symptomatology

The self-report version of the Inventory of Depressive Symptomatology (IDS-SR; Dutch translation Altrecht GGZ [52, 65]) consists of 30 items that are scored on a four-point scale. The total score ranges from 0 to 84. The IDS-SR has acceptable psychometric properties and it is a treatment-sensitive measure in depressed outpatients [65–67].

#### Secondary outcome measures

##### Functional impairment: Sheehan disability scale

The Sheehan Disability Scale (SDS [68], Dutch translation) measures functional impairments that are caused by symptoms. Three ten-point scales measure to what extent symptoms impair function in three domains. The sum score, which ranges from 0 (unimpaired) to 30 (highly impaired), gives an overall impression of the level of impairment experienced by the patient. Leon et al. [69] report high internal consistency. Their analyses also provide empirical support for construct validity.

##### Positive mental health: mental health continuum-short form

The Mental Health Continuum-Short Form (MHC-SF, Dutch translation [70]) comprises 14 items. The occurrence of various types of feelings of well-being in the last month are rated on a six-point Likert scale. The total score on the scale can range from 0 to 70 points. Higher scores indicate a higher level of well-being. High internal and convergent validity and moderate test-retest reliability have been reported [70].

#### Potential predictors

##### Demographic variables

Patients complete a short questionnaire regarding a number of demographic variables regarding gender, age, cultural background, marital status, education, employment status and income.

##### Traumatic life-events (including recent): list of threatening experiences

The List of Threatening Experiences [71, 72] (Dutch translation) consists of descriptions of 21 life events and an assessment of occurrence (yes or no) before and after the sixteenth year of life and during the past twelve months. The checklist has shown to have high test-retest reliability and good agreement with informant information [71, 73].

##### Life events (lifetime): checklist life events for DSM-5

The Checklist Life Events for DSM-5 (LEC-5) [74], Dutch translation [75]) is a self-report measure that assesses lifetime exposure to 16 potentially traumatic events. Different levels of exposure (on a six-point nominal scale) can be indicated by the respondent. Psychometrics are not available for the LEC-5 but the similar LEC for DSM-IV has been shown to have adequate psychometric properties [76]. Given the minimal revisions by comparison with the original version of the LEC, few psychometric differences with the latest version are expected.

##### Symptoms: brief symptom inventory

The Brief Symptom Inventory (BSI [77], Dutch translation [78]) is a brief psychological self-report symptom scale with good psychometric properties. It consists of 53 items that are scored on a five-point scale. The BSI shows the extent to which the person has suffered from psychological and / or physical symptoms during the past period. The BSI has nine subscales: Somatisation (SOM), Obsessive-Compulsive (O-C), Interpersonal Sensitivity (I-S), Depression (DEP), Anxiety (ANX), Hostility (HOS), Phobic Anxiety (PHOB), Paranoid Ideation (PAR), and Psychoticism (PSY). The measure also provides a score for the total number of complaints, total symptoms and symptom severity.

##### Agoraphobia: the agoraphobia scale

The Agoraphobia Scale (AGO, [79]) consists of 20 items concerning agoraphobic situations. These are rated for anxiety/discomfort (0–4) and for avoidance (0–2). The AGO is a valid and reliable instrument [79].

##### Self-esteem: Rosenberg self-esteem scale

The Rosenberg Self-Esteem Scale (RSES [80]; Dutch translation [81]) is designed to measure self-esteem. The RSES consists of 10 items that are scored on a 4-point scale ranging from “completely agree” to “completely disagree”. The RSES has good psychometric properties [81, 82].

##### Rumination: rumination response scale

The Rumination Response Scale (RRS [83]; Dutch translation [84]) is designed to measure ruminative thoughts and behaviours in patients with MDD. It consists of 22 items that are scored on a four-point scale ranging from “almost never” to “always”. The RRS has good psychometric properties [83].

##### Defence mechanisms: defense style questionnaire

The Defense Style Questionnaire (DSQ [85], Dutch translation [86]) is a self-report inventory that measures specific defence mechanisms. The DSQ consists of forty items in a nine-point Likert format that derive scores for twenty defence mechanisms, two items for each. These mechanisms are organised as four sub-factors (immature, mature, image-distorting and neurotic). The DSQ has proven to be a valid and reliable instrument.

##### Personality organisation: inventory of personality organisation

The Inventory of Personality Organisation (IPO [87], Dutch translation [88]) is a self-report instrument intended to measure a patient’s level of personality organisation. The IPO is a 57-item self-report questionnaire with three scales, each relevant to a different dimension of Kernberg’s personality organisation model. All items are rated on a five-point Likert-scale format ranging from 1 (never true) to 5 (always true). Adequate internal consistency and good test-retest reliability have been reported [89].

#### Alliance, therapist characteristics and allegiance

##### Working Alliance: working Alliance Inventory-12

The client version of the Working Alliance Inventory (WAI [90], Dutch Translation [91]) measures the quality of the therapeutic relationship as perceived by the patient. The WAI-12 is a shortened version for measuring the therapeutic relationship and it consists of twelve items, each item being judged on a five-point scale (1 = rare or never, 5 = always). In 2009, the WAI-12 was validated for the Flemish population and the three-factor structure of the WAI-12 was confirmed [92].

##### Therapist characteristics: Counsellor rating form-short version (CRF-S)

The CRF-S [40] measures the reliability and expertise of the therapist as perceived by the patient. The reliability of the condensed version (CRF-S [41]) appeared to be comparable to the full version. The shortened CRF consists of 12 items, with all items scored on a seven-point rating scale ranging from “not very” to “very”. Due to the absence of an existing Dutch translation, the CRF-S was translated into Dutch for the current study. The translation procedures of Beaton et al. [93] were followed for this purpose.

##### Allegiance

Allegiance is measured using a questionnaire that is filled in by the therapists. It contains 14 questions, that are rated on a 5-point Likert scale, for example about the number of years of experience with the particular method, the amount of training followed in the respective treatment method, the therapists willingness to further develop oneself as a CBT or SPSP therapist through training or supervision, if the therapist visits specific congresses or meetings pertaining to the particular treatment method and to what extent he or she has confidence in the effectiveness of the particular treatment. The higher the score, the higher the indicated degree of allegiance. Also a question with regard to the general allegiance of a practitioner is included. The questionnaire is based on recommendations by Leykin and DeRubeis [51], which are partly based on research by Luborsky et al. [48, 94].

#### Treatment integrity

About 40 therapists are expected to take part in the study. The exact number will depend on various circumstances such as the time it takes to include all patients and the therapists turnover rate in the locations. The minimum requirement to participate as a therapist in the study is a master’s degree in medicine or psychology. Therapists cannot start treating patients included in the study until they have received a three-day course in SPSP and a three-day course in CBT for depression that are given by qualified and experienced experts. Audio recordings are made of every treatment session to ensure and measure treatment integrity. Recordings are discussed in the presence of peers during supervision that is given by trained supervisors every 2 weeks. Training and supervision sessions for SPSP are therefore based on the Handbook of Short-Term Psychoanalytic Supportive Psychotherapy [60]. CBT training is also based on an existing Dutch CBT protocol [63]. There is no scale for measuring treatment integrity for SPSP and within this study we will develop and test an instrument that measures the most important method specific elements of SPSP, namely adequate psychoanalytic support and the different levels of discourse. Adherence in CBT was measured with the Cognitive Therapy and Adherence Scale (CTACS) [95]. The CTACS measures competence as well as adherence for CBT in general. It has been extensively studied in patients suffering from cocaine dependence but was designed and is appropriate for measuring both competence and adherence for all types of CBT. It has good internal reliability and acceptable interrater reliability [95]. The criterion validity of the scale is high [96, 97]. In the current study, the applicability of the CTACS will be studied specifically for depressed patients.

#### Other assessments

The Mini-international neuropsychiatric interview (MINI [57]), a structured interview for classifying DSM-IV and DSM 5 disorders, is used to assess whether the criteria for a Major Depressive Episode have been met. Other psychiatric classifications are also assessed during the interview. The psychometric qualities of the MINI have been reported to be good.

A record is made for all patients of whether they are using medication at the start of treatment and whether there are any changes in the prescribed medication during the 8-week treatment period.

### Statistical analysis

#### Establishing non-inferiority

The study compares the effectiveness of two forms of treatment (SPSP and CBT). The primary dependent variable is the average post-treatment score for the two groups on the IDS-SR. An ANCOVA with treatment type received as a fixed effect and baseline depression scores measured on the IDS-SR as a covariate will be performed to calculate the mean and 95% CI of the difference between the groups at the final time point on the IDS-SR. This analysis forms the basis for demonstrating that SPSP reduces depressive symptoms at least as effectively as CBT. If the lower limit of the 95% CI is above the non-inferiority limit of − 5 on the IDS-SR, it will be assumed that SPSP is not inferior to CBT. Non-inferiority analysis will be performed on the basis of both per protocol and intention-to-treat analysis.

Additional multilevel repeated measures will be used to further analyse changes in depressive symptoms and the other secondary outcome measures (functional impairment and positive mental health) and to examine possible differences between both treatment groups over time. If there are significant differences between groups in medication use or other potentially confounding variables, these variables will be included as covariates.

#### Identifying and building a model for predictive and prescriptive factors

Using the Personalised Advantage Index (PAI) approach, we will investigate which form of therapy works optimally for subgroups of depressed patients [27]. The aim is to build and validate a predictive model using machine learning techniques that combines multiple predictors and moderators to make predictions regarding the optimal, expected effectiveness of treatment for a specific patient on the basis of the interaction between patient characteristics and the type of treatment [24]. If the PAI actually has predictive value, it can be expected that patients who by chance receive the treatment method that does not match the PAI score in phase one are the ones that will benefit most from a switch of treatment method. Exact statistical procedures regarding PAI development are beyond the scope of this study protocol and they will be discussed in the relevant publications.

#### The effect of therapist or therapy change after initial non-response

The effect of different follow-up treatment strategies, after an initial non-response to treatment (defined as an improvement of less than 50% over the initial measurement on the IDS-SR), on depressive symptoms, functional impairment and positive mental health will be analysed using multilevel repeated-measures linear-mixed modelling with group, time and group × time interaction as fixed factors.

#### Moderator and mediator analysis

Most moderator and mediation analysis regarding allegiance and adherence will be performed with regression-based methods [98]. Additionally, cross-lagged panel analysis will be used to investigate the directionality and reciprocity of the relations between working alliance (WAI) and symptoms (IDS-SR) over time [99, 100]. We will also study whether a cut-off score for the working alliance can be determined that is predictive of a significantly lower treatment result by means of an receiver operating characteristic analysis. In line with Zilcha-Mano [101] the relative speed of change in the quality of the working alliance for each patient will also be explored in an additional analysis as a possible factor that influences outcome since, in her theory, some patients are better equipped to benefit from the therapeutic relationship.

## Discussion

The overarching purpose of the study described in this protocol is to acquire knowledge that can help increase the effectiveness of psychotherapy for depression in clinical practice. We aim to do this by addressing a number of research questions in one study with a comprehensive design. Some of these questions have never been the direct focus of a research project. By enrolling depressed outpatients who are randomised to two active treatment conditions (CBT and SPSP), we start the study with a non-inferiority design. This design enables us to address multiple issues. We are able to explore whether SPSP is non inferior to CBT, which extends the evidence base of psychodynamic psychotherapy for depression, which is frequently applied but still relatively understudied. We hope the study will also reveal prescriptive and prognostic factors for treatment response. We will try to build and validate a model which enables us to predict which treatment works best for whom, in other words to personalise treatment. Developing personalised treatment selection strategies is a very promising way to increase the effectiveness of psychotherapy. In the D*Phase study, prediction based on PAI in the first phase of the study can be validated on the basis of the treatment results in phase two of the study.

A novel aspect of the design is found in the second part of the study, which focuses on patients who do not benefit (or who do not benefit enough) from psychotherapy. This is often the case in clinical practice. These patients are randomised again into three groups (continuing the initial treatment with the same therapist, continuing the initial treatment with another therapist and continuing the other type of treatment with another therapist). This provides us with opportunities to find evidence for the expert-based advice given in practice guidelines for the treatment of MDD. The guidelines tell us to switch to another kind of therapy when not enough progress is made but there is no empirical evidence for this advice. Furthermore, we explore what happens when patients switch to another therapist. This is particularly interesting given the assumption made by some researchers that the effects of psychotherapy largely depend on common factors.

By incorporating questionnaires on the working alliance and therapist characteristics that are completed at different points in time, we hope to gain insight into the influence of the working alliance on the effect of psychotherapy. The repeated measures design will provide an opportunity to evaluate whether symptom change occurs in advance of, and is related to, the working alliance. This is one of only a few studies this temporal effect. We also hope to shed light on whether, and which, characteristics of the therapist have an effect on the working alliance and we will address the topic of the relevance of therapist allegiance, with the treatment methods studied, as a predictor of treatment outcome.

The current study is distinctive in the sense that it provides an opportunity to explore the influence of an important common factor (the alliance) and also therapy-specific factors (treatment differences and treatment integrity) on treatment effect in a single, large cohort. Treatment integrity is adequately measured for both conditions. The study also provides several openings for post-hoc analysis. For example, if we find that the therapy and/or therapist switch has an effect, we will be in a position to determine which factors contribute to a possible effect of the switch. If there should indeed be a relationship between competence/adherence and treatment effect, this could be a cautious indication that method-specific interventions *do* matter in psychotherapy. Of course, findings will only remain global indications and there is no way to measure all possible factors that could play a role.

We therefore intend to implement a pragmatic, methodically strong, study in which the naturalistic setting will contribute to the generalisability of the results to everyday practice. This is a challenging undertaking and it also has certain limitations. Due to the naturalistic setting, for instance, it is not possible to control completely for the use of medication, although the use of medication is monitored and, if necessary, statistically controlled for in the analysis. Different treatment options for depression are available in the treatment centres and patients can choose a different kind of treatment offered by the treatment centre and refused to participate in the research project. This may result in a bias. Secondly, a double-blind design is never possible in a psychotherapy study because the therapist and patient are obviously aware of the kind of treatment that is being administered. However, by digitalising the administration of all the questionnaires that are used, both researchers and therapists are blinded to all patient-reported results and researchers are blinded to the treatment conditions. Thirdly, in spite of the fact that we include several potential predictors, one possible mediator and several moderators, there will certainly be important factors (latent and otherwise) that are not assessed in the current study. Lastly, because the study is primarily powered for the non-inferiority phase, it is expected that phase two of the study only has sufficient power to detect medium to large differences between the groups. Despite this relatively limited power, we still expect this phase of the study will generate meaningful observations on the effects of therapist and treatment switching.

The therapists in the study are thoroughly trained and supervised in the treatment methods concerned in order to ensure that the non-inferiority part of the study will not be biased too much by differences in therapist competence with respect to the different treatment methods. However, most therapists are expected to have more experience with CBT. We will be able to take treatment experience into account as a confounder in our statistical analyses. Furthermore, the measurement of treatment allegiance and treatment integrity will allow for statistical correction for confounding by these factors, at least when it proves to be relevant to do so. There is no instrument to measure treatment integrity for SPSP and so it has to be developed during the course of the study.

Despite these limitations, the design of this study, which compares two different treatments head-to-head, means it will have implications at different levels. In addition to enhancing the evidence base for SPSP, it can teach us more about prescriptive factors for different treatments for MDD and shed light on the role of the therapist in terms of maximising treatment effect by studying the working alliance and the effect of treatment integrity and allegiance. On top of that, this study is the first to manipulate the therapist as a factor through a switch of psychotherapist when therapy does not have the intended effect on depressive symptoms. In conclusion, we hope to provide a large number of new insights that will help to increase the effectiveness of psychotherapy and contribute to the improvement of health care for adults suffering from MDD.

## Data Availability

The datasets used and/or analysed during the current study are available from the corresponding author on reasonable request.

## Abbreviations

AGO: Agoraphobia Scale
ANCOVA: Analysis of covariance
APS: Adequate Psychoanalytic Support
BSI Brief: Symptom Inventory
CBT: Cognitive behaviour therapy
CI: Confidence interval
CRF-S: Counsellor Rating Form Short Version
DSM-IV: Diagnostic and Statistical Manual-IV
DSM 5: Diagnostic and Statistical Manual 5
DSQ: Defence Style Questionnaire
DALYs: Disability-adjusted life years
IPO: Inventory of Personality Organization
IDS-SR: Inventory of Depressive Symptomatology – Self-Report
LEC-5: Life Events Checklist for DSM 5
LTE: List of Threatening Experiences
METC: Medical Ethics Board
MDD: Major Depressive Disorder
MHC-SF: Mental Health Continuum - Short Form
MINI: Mini-international neuropsychiatric interview
PAI: Personalised Advantage Index
SPSP: Psychodynamic Supportive Psychotherapy
RRS: Rumination Response Scale
RSES: Rosenberg Self-Esteem Scale
SDS: Sheehan Disability Scale
WAI: Working Alliance Inventory
